# Annual Mortality Rate and Causes of Death in a Hemodialysis Unit at a Rural Community Hospital in Chile: A Retrospective Cohort Study

**DOI:** 10.7759/cureus.105267

**Published:** 2026-03-15

**Authors:** Lucas Romero Vinet, Romeo Leiva Espinoza, Marcela Pérez Rodriguez

**Affiliations:** 1 Hemodialysis Unit, Hospital San Pedro de Los Vilos, Los Vilos, CHL

**Keywords:** cardiovascular mortality, chronic hemodialysis, community hospital, mortality rate, rural health, vascular access

## Abstract

Introduction: Mortality among patients receiving chronic hemodialysis remains substantially higher than in the general population, with cardiovascular and infectious diseases as the leading causes of death. Data from rural, low-complexity community hospitals are limited. In geographically remote areas, factors such as long distances to higher-complexity referral centers and potential delays in access to specialized care may influence patient outcomes. This study aimed to determine the annual mortality rate and causes of death in a cohort of chronic hemodialysis patients treated at a rural community hospital in Chile.

Methods: We conducted a retrospective cohort study including all adult patients receiving maintenance chronic hemodialysis at our center between January 1, 2025, and January 31, 2026, corresponding to a 13-month observation period. Transient patients who were temporarily dialyzed at our unit for less than two months before returning to their original dialysis center were excluded. Total patient-years were calculated, and mortality was expressed as deaths per 100 patient-years with 95% confidence intervals.

Results: A total of 59 patients were included (mean age 65 ± 14.7 years; 69.4% male). During follow-up, 13 patients died, accounting for 53.45 patient-years. The overall mortality rate was 24.3 deaths per 100 patient-years (95% CI: 13.0-41.6). Cardiovascular causes accounted for 61.5% of deaths, infectious causes for 30.7%, and traumatic causes for 7.6%. Six patients initiated dialysis during the study period; in this subgroup, the mortality rate was 56.5 deaths per 100 patient-years.

Conclusion: The observed mortality rate was slightly higher than rates reported in large international registries. Although limited by the small number of patients in a single rural dialysis unit, these findings underscore the importance of continued outcome monitoring in rural dialysis units and suggest that structural and geographic factors may influence patient survival.

## Introduction

Despite advances in dialysis therapy and comprehensive management of comorbidities, survival among patients with end-stage kidney disease receiving maintenance hemodialysis remains limited, with mortality rates significantly higher than in the general population [1].

According to international registries, annual mortality among patients receiving hemodialysis shows important geographic variation. In the United States, reported rates are approximately 15-18% per year [2], while in Western Europe, mortality ranges between 10% and 15% [3]. In contrast, Japan reports some of the lowest annual mortality rates worldwide, around 9-10% [4].

Data regarding dialysis outcomes in Latin America remain limited and heterogeneous. In Brazil, which has one of the largest hemodialysis populations worldwide, the reported crude annual mortality rate is approximately 19.5%, although available data are often derived from large dialysis networks or voluntary registry reports [5]. These findings highlight the need for additional studies describing outcomes in different healthcare settings, particularly in smaller or rural dialysis units.

Several studies have demonstrated that mortality risk is significantly higher during the first months after dialysis initiation, particularly within the first 90 days, a period characterized by greater clinical instability and higher comorbidity burden. Cardiovascular events, including myocardial infarction, heart failure, and stroke, represent the leading cause of death, followed by infections, particularly those related to vascular access. Withdrawal from dialysis and malignancy account for a smaller proportion of deaths [1,6].

In Chile, the reported crude annual mortality rate among patients undergoing chronic hemodialysis is approximately 10.9%, comparable to some Western European countries [7].

Hospital San Pedro de Los Vilos is a low-complexity community hospital located in the Coquimbo region of Chile, approximately 190 kilometers from the nearest tertiary referral center. Geographic distance from higher-complexity facilities may influence timely access to specialized management of acute complications, potentially impacting patient outcomes.

The aim of this study was to determine the overall and cause-specific mortality rate among patients receiving chronic hemodialysis at this rural community hospital during the 2025-2026 period, expressed as deaths per 100 patient-years of follow-up, and to describe mortality according to vascular access type.

## Materials and methods

Study design

This was a retrospective observational study conducted at the Hemodialysis Unit of Hospital San Pedro de Los Vilos, located in the Coquimbo region of Chile. The study period extended from January 1, 2025, to January 31, 2026.

Study population

The study population included all adult patients assigned to the Hemodialysis Unit of Hospital San Pedro de Los Vilos who were receiving thrice-weekly maintenance high-flux hemodialysis during the study period.

Transient patients, defined as individuals temporarily dialyzed at our center for less than two months before returning to their original dialysis unit, were excluded due to incomplete follow-up data.

Incident patients were defined as those initiating chronic hemodialysis during the study period, whereas prevalent patients were those already receiving dialysis at the beginning of the observation period.

Data collection

Causes of death were obtained from official death certificates issued by the Chilean Civil Registry. When the cause of death recorded on the death certificate was nonspecific (e.g., cardiorespiratory arrest or multi-organ failure), additional information from emergency department records, the patient’s clinical chart, and imaging reports from computed tomography (CT) scans performed at referral centers was reviewed to determine the most appropriate underlying cause. Deaths recorded with nonspecific causes, such as cardiorespiratory arrest or multi-organ failure, were classified as sudden death and categorized as cardiovascular events.

The following variables were recorded: age, sex, type of vascular access (arteriovenous fistula or tunneled central venous catheter), date of dialysis initiation, date of last follow-up, and date and cause of death as documented on the death certificate.

Statistical analysis

Total patient-years were calculated by summing individual time at risk from either the beginning of the study period or the date of dialysis initiation (whichever occurred later) until death or the end of follow-up. Mortality rates were expressed as deaths per 100 patient-years. Mortality rates were expressed as deaths per 100 patient-years to account for differences in individual follow-up time and the inclusion of both incident and prevalent patients. Ninety-five percent confidence intervals were estimated assuming a Poisson distribution.

Given the higher mortality risk during the first 90 days after dialysis initiation, a subgroup analysis was performed comparing incident and prevalent patients. A sensitivity analysis excluding events and person-time occurring within the first 90 days of dialysis initiation was also conducted.

Due to the descriptive nature of the study and the limited sample size, no hypothesis testing was performed and no p-values were calculated. Consequently, multivariable analyses were not conducted. Statistical analyses were performed using Microsoft Excel (Microsoft Corp., Redmond, WA, USA).

Ethical considerations

This retrospective study used anonymized clinical data collected during routine clinical practice. No identifiable patient information was included.

## Results

Baseline characteristics

Between January 1, 2025, and January 31, 2026, a total of 59 patients received maintenance hemodialysis at our center. The mean age was 65.3 ± 14.7 years (range: 30-92), and 41 patients (69.4%) were male. At the end of the study period, 34 patients (57.6%) were using an arteriovenous fistula and 25 (42.4%) a tunneled central venous catheter. Six patients (10.2%) initiated hemodialysis during the study period.

Baseline characteristics of the cohort are summarized in Table 1.

**Table 1:** Baseline characteristics of the hemodialysis cohort (n = 59) Data are presented as n (%) or mean ± standard deviation (SD).

Variable	n (%) or mean ± SD
Total number of patients	59
Age, mean ± SD (years)	65.3 ± 14.7
Male sex	41 (69.4%)
Arteriovenous fistula	34 (57.6%)
Hemodialysis catheter	25 (42.4%)
Incident patients	6 (10.2%)

Mortality outcomes

During follow-up, 13 patients died, corresponding to a crude mortality proportion of 22.0% (13/59). The total accumulated follow-up time was 53.45 patient-years, resulting in an overall mortality rate of 24.3 deaths per 100 patient-years (95% CI: 13.0-41.6).

Among incident patients, two deaths occurred during 3.54 patient-years of follow-up, corresponding to a mortality rate of 56.5 deaths per 100 patient-years (95% CI: 6.8-204.3).

At the time of death, seven patients (53.8%) were using a tunneled central venous catheter and six (46.2%) an arteriovenous fistula.

Overall and incident mortality outcomes are summarized in Table 2, and the distribution of vascular access among deceased patients is presented in Table 3.

**Table 2:** Overall and incident mortality in chronic hemodialysis Data are presented as n (%), mortality rates per 100 patient-years, and 95% confidence intervals (CI). Patient-years: cumulative individual follow-up time expressed in years.

Variable	Result
Deaths during follow-up, n (%)	13 (22.0%)
Total patient-years	53.45
Mortality rate (per 100 patient-years)	24.3 (95% CI: 13.0–41.6)
Incident patients, n (%)	6 (10.2%)
Deaths among incident patients, n (%)	2 (33.3%)
Incident patient-years	3.54
Mortality rate in incident patients (per 100 patient-years)	56.5 (95% CI: 6.8–204.3)

**Table 3:** Type of vascular access among deceased patients (n = 13) Data are presented as n (%).

Type of vascular access	n (%)
Arteriovenous fistula	6 (46.2%)
Tunneled central venous catheter	7 (53.8%)

Causes of death

Cardiovascular causes accounted for eight deaths (61.5%), infectious causes for four (30.7%), and traumatic causes for one (7.6%). One patient died after dialysis withdrawal following a hip fracture after shared decision-making with the treating medical team.

In another case, a patient presented with severe hyperkalemia requiring urgent renal replacement therapy. Transfer to a tertiary center for acute dialysis was not feasible due to hemodynamic instability at the time of evaluation.

Detailed causes of death and their classification are presented in Table 4.

**Table 4:** Detailed causes of death among patients receiving chronic hemodialysis AVF, arteriovenous fistula; CVC, tunneled central venous catheter ^a^When the cause of death recorded on the death certificate was nonspecific (e.g., cardiorespiratory arrest or multiorgan failure), additional information from emergency department records or the patient’s clinical chart was reviewed to determine the most appropriate classification. ^b^One patient died after withdrawal from dialysis following a hip fracture, after shared decision-making between the patient and the treating medical team. ^c^Another patient presented with severe hyperkalemia requiring urgent renal replacement therapy. Transfer to a tertiary center for acute dialysis was not feasible due to hemodynamic instability at the time of evaluation.

Cause of death	Place of death	Classification	Vascular access	Age (years)
Sudden death^a^	Out-of-hospital	Cardiovascular	Tunneled CVC	75
Acute pulmonary edema/hyperkalemia^c^	In-hospital	Cardiovascular	AVF	84
Community-acquired pneumonia	In-hospital	Infectious	Tunneled CVC	87
Sudden death^a^	Out-of-hospital	Cardiovascular	Tunneled CVC	80
Acute pyelonephritis	In-hospital	Infectious	AVF	74
Sepsis of abdominal origin	In-hospital	Infectious	AVF	79
Sudden death^a^	In-hospital	Cardiovascular	Tunneled CVC	71
Intraparenchymal hemorrhage/hypertensive emergency	In-hospital	Cardiovascular	AVF	46
Hip fracture (dialysis withdrawal)^b^	Out-of-hospital	Trauma	Tunneled CVC	83
Hypovolemic shock/ruptured thoracoabdominal aneurysm	In-hospital	Cardiovascular	AVF	57
Refractory hyperkalemia	In-hospital	Cardiovascular	Tunneled CVC	78
Sudden death^a^	Out-of-hospital	Cardiovascular	Tunneled CVC	83
Septic shock/healthcare-associated pneumonia	In-hospital	Infectious	AVF	77

**Figure 1:**
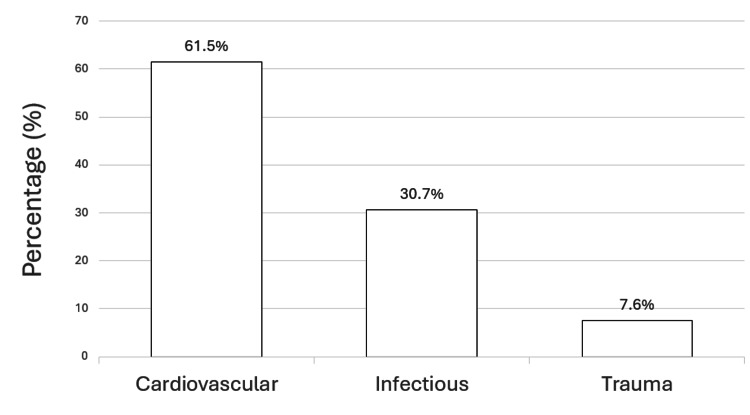
Cause-specific mortality distribution Data are presented as percentages (%) of total deaths among patients receiving chronic hemodialysis (n = 13) during follow-up.

The distribution of causes of death is illustrated in Figure 1.

## Discussion

In this series of patients receiving chronic hemodialysis, the observed mortality rate was 24.3 deaths per 100 patient-years during the study period. Mortality among patients undergoing hemodialysis varies considerably across regions depending on demographic characteristics, comorbidity burden, and healthcare system factors. In Latin America, available data remain limited and heterogeneous. For example, a large Brazilian cohort reported a crude annual mortality rate of approximately 19.5% [5], while in Chile, the reported crude annual mortality among patients receiving chronic hemodialysis is approximately 10.9% [7]. Although the mortality observed in our cohort appears higher than these national estimates, the small number of patients evaluated (n = 59) should be considered when interpreting these results.

In small populations, minor variations in the absolute number of events can substantially influence the overall mortality rate, leading to wide statistical fluctuations and broad confidence intervals. In this context, the occurrence of one or two additional deaths may significantly alter the annual estimate, limiting direct comparisons with large national or international registries.

Another relevant factor is the geographic setting of our center. Hospital San Pedro de Los Vilos is located at a considerable distance from higher-complexity referral hospitals. The nearest facility with access to advanced critical care support and broad-spectrum intravenous antibiotics is approximately one hour away by ambulance, while the closest center capable of providing emergency hemodialysis is located in Ovalle, approximately two and a half hours away. These geographic constraints may contribute to delays in the management of severe infections, refractory hyperkalemia, and other dialysis-related emergencies requiring urgent renal replacement therapy.

In such settings, access to urgent renal replacement therapy often depends on inter-hospital transfer to tertiary centers. In our cohort, patients presenting with severe hyperkalemia required prolonged stabilization in the emergency department while awaiting transfer for definitive treatment. In one case, transfer to a higher-complexity hospital occurred more than 12 hours after the initial evaluation, illustrating the logistical challenges faced by rural dialysis programs when managing life-threatening metabolic complications.

The distribution of causes of death showed a predominance of cardiovascular events, consistent with international literature in which cardiovascular disease represents the leading cause of mortality among patients undergoing hemodialysis [1,5]. Infectious causes also accounted for a substantial proportion of deaths, reinforcing the importance of preventive strategies, early detection, and timely management of infections in this vulnerable population.

In our series, a relatively high proportion of patients were using tunneled central venous catheters at the time of death. Catheter use has been consistently associated with increased infection risk and higher mortality in patients undergoing hemodialysis [6], which may partially contribute to the distribution of causes of death observed in this study.

Finally, mortality among incident patients was higher than in the overall cohort. Although the estimate is associated with a wide confidence interval due to the limited number of patients, this finding aligns with previous reports describing increased mortality during the first months after dialysis initiation [1,6]. This early high-risk period has been attributed to greater clinical instability, unplanned dialysis initiation, and more frequent use of central venous catheters.

Limitations 

This study has several limitations. First, the sample size was small and derived from a single center, which limits external generalizability. Second, the retrospective design may be subject to residual confounding and information bias. Third, although causes of death were determined using official death certificates and complemented by review of available hospital medical records, complete clinical information was not available for patients who died at other institutions, which may have led to some degree of misclassification. Although comorbidity information was available in the medical records, these variables were not included in the present analysis due to the descriptive design and the limited sample size of the cohort.

## Conclusions

In this cohort of patients receiving chronic hemodialysis at a low-complexity community hospital, the observed mortality rate was slightly higher than those reported in international registries. The distribution of causes of death was consistent with prior literature, with cardiovascular and infectious events representing the predominant contributors. The small sample size limits the generalizability of these findings and may influence the stability of annual mortality estimates.

These results highlight the importance of continued outcome surveillance in small dialysis programs and may have implications for healthcare planning in rural settings, particularly regarding timely access to specialized nephrology care and emergency services in geographically remote areas.
